# Lack of the pattern recognition molecule mannose-binding lectin increases susceptibility to influenza A virus infection

**DOI:** 10.1186/1471-2172-11-64

**Published:** 2010-12-23

**Authors:** Wei-Chuan Chang, Mitchell R White, Patience Moyo, Sheree McClear, Steffen Thiel, Kevan L Hartshorn, Kazue Takahashi

**Affiliations:** 1 Program of Developmental Immunology, Department of Pediatrics, Massachusetts General Hospital, Harvard Medical School, Boston, MA 02114, USA; 2 Department of Medicine, Boston University School of Medicine, Boston, MA02118, USA; 3 Department of Medical Microbiology and Immunology, Aarhus University, DK-8000 Aarhus, Denmark

## Abstract

**Background:**

Mannose-binding lectin (MBL), a pattern recognition innate immune molecule, inhibits influenza A virus infection *in vitro*. MBL deficiency due to gene polymorphism in humans has been associated with infection susceptibility. These clinical observations were confirmed by animal model studies, in which mice genetically lacking MBL were susceptible to certain pathogens, including herpes simplex virus 2.

**Results:**

We demonstrate that MBL is present in the lung of naïve healthy wild type (WT) mice and that MBL null mice are more susceptible to IAV infection. Administration of recombinant human MBL (rhMBL) reverses the infection phenotype, confirming that the infection susceptibility is MBL-mediated. The anti-viral mechanisms of MBL include activation of the lectin complement pathway and coagulation, requiring serum factors. White blood cells (WBCs) in the lung increase in WT mice compared with MBL null mice on day 1 post-infection. In contrast, apoptotic macrophages (MΦs) are two-fold higher in the lung of MBL null mice compared with WT mice. Furthermore, MBL deficient macrophages appear to be susceptible to apoptosis *in vitro*. Lastly, soluble factors, which are associated with lung injury, are increased in the lungs of MBL null mice during IAV infection. These results suggest that MBL plays a key role against IAV infection.

**Conclusion:**

MBL plays a key role in clearing IAV and maintaining lung homeostasis. In addition, our findings also suggest that MBL deficiency maybe a risk factor in IAV infection and MBL may be a useful adjunctive therapy for IAV infection.

## Background

IAV is an enveloped RNA virus with a capsule that contains neuraminidase and hemagglutinin, both of which are glycosylated [1]. One of the characteristics of IAV infection is the production of a large number of apoptotic cells [2]. IAV infection, a very common infection, is estimated to cause 20 fatalities and 52 hospitalizations per 100,000 persons in the United States alone [3].

The first line of host defense mechanism is the innate immunity. The innate immune system utilizes innate immune cells, including phagocytes, such as macrophages (MΦs) and polymorphonuclear neutrophils (PMNs) [4]. In the innate immune system, pathogens are identified by pattern recognition molecules, including lectins [4]. One such lectins is MBL, a serum protein, which is primarily synthesized in the liver [5]. MBL was identified to be a β-inhibitor that neutralized IAV in a calcium-dependent fashion [6,7]. A genetic basis for MBL deficiency correlating with infection susceptibility was established in the 1990s [8]. Many *in vitro *studies have described MBL's anti-viral functions, including viral aggregation, inhibition of viral hemagglutination and opsonization of virus [7,9,10]. MBL also activates complement via the lectin pathway, which requires MBL-associated serine proteases (MASPs) [11]. The lectin complement pathway, therefore, does not require antibody, which is not immediately available since the adaptive immune system has not had time to respond at the moment of initial viral infection. A complex of MBL and MASP also initiates coagulation via thrombin-like activity [12,13]. Coagulation is a primitive yet an effective host defense mechanism. For example, tachylectins in horseshoe crab hemolymph provide innate immune protection by clotting lipopolysaccharide and β-glucan [14] (PAMPs of Gram negative bacteria and fungi, respectively).

MBL belongs to the collectin family that is characterized structurally by a type II collagen-like domain at the C-terminus followed by a neck region and a carbohydrate recognition domain (CRD) at the N-terminus [15]. The collectin family also includes lung surfactant protein (SP)-A and SP-D [15]. These surfactant proteins provide lung protection and are constitutively present in lungs, where initial IAV infection typically takes place [16]. Mice lacking SP-A or SP-D have increased susceptibility to IAV infection [17]. In contrast, MBL has not been detected in healthy lungs although MBL levels increase in the lung following infection [18] and a messenger RNA for MBL has been detected at very low levels in the lung [5]. Nonetheless, MBL deficiency has been associated with lung disease, including non-cystic fibrosis [19-21].

Humans have a single MBL protein derived from a single gene while mice have two proteins, termed MBL-A and MBL-C that are transcribed from two independent genes located on different chromosomes [15]. Human MBL is genetically homologous to MBL-C although MBL-A is functionally similar to human MBL in that these two proteins are both acute phase proteins while mouse MBL-C is constitutively expressed [15]. The human MBL gene has multiple polymorphisms, some of which produce low levels of MBL and dysfunctional MBL and have been clinically associated with susceptibility to infection [15]. The clinical observations were confirmed in animal models of infection using mice that lack both MBL-A and MBL-C and which are therefore null for MBL[22].

In this study, we investigated whether MBL plays a role against IAV infection by comparing MBL null and WT mice and further analyzed anti-viral mechanisms of MBL.

## Methods

### MBL binding assay

This assay was performed using previously described methods with a minor modification [23]. Briefly, 96 well plates were coated with viruses (1000^-1 ^HA titer/well) in 50 μl of a bicarbonate buffer, pH 9.5 and blocked and then various concentrations of recombinant human MBL (rhMBL) in 50 μl was added and incubated at room temperature. After washing, virus-bound MBL was detected by mouse anti-hMBL monoclonal Ab (mAb) 131-1 (State Serum Institute, Denmark) followed by alkaline-phosphatase conjugated goat anti-mouse IgG Ab and pNTP substrate. The reactions were read at 415 nm using a SpectraMax M5 (Molecular Devices, CA). For mannan inhibition experiments, virus coated wells were incubated with various concentrations of mannan together with rhMBL at 2 μg/ml. Binding was expressed as the OD 415 nm reading and mannan binding inhibition was calculated as % Inhibition = [(OD 415 without mannan) - (OD 415 nm with mannan))/(OD 415 nm without mannan)] × 100.

### Virus neutralizing assay in vitro

Test samples included rhMBL, purified MBL-A, purified MBL-C, and serum from WT mice and various mice lacking MBL-A, MBL-C or both. Purified mouse MBLs, rhMBL and sera were used at the indicated concentrations. The assay was performed as previously described [24]. Briefly, viruses were pre-incubated with test samples, washed and then incubated with Madin-Darby canine kidney (MDCK) cells. Infection was assayed by FITC-conjugated anti-IAV antibody (Ab)(Millipore, MA). Fluorescent foci were counted. Virus neutralizing activity (%) was calculated by the formula: (fluorescent foci counts (FFC) in saline - FFC in test sample) × 100/FFC in saline control.

### Assays of lectin complement activity and thrombin-like activity

The lectin pathway assay was performed with a minor modification of a previously described method [23]. Briefly, 96 well plates were coated with IAV in bicarbonate buffer, pH 9.5. After washing and blocking with BSA, wells were incubated with 1% serum diluted in a binding buffer, 10 mM Tris, pH 7.4, 10 mM CaCl2, 1 M NaCl and incubated at room temperature. After washing, the wells were incubated with human C4 at 37°C. After washing again, the wells were incubated with rabbit anti-hC4c Ab followed by alkaline phosphatase-conjugated goat anti-rabbit IgG Ab and then with pNTP. The plates were read at OD 415 nm. Pooled human serum with a known MBL concentration (State Serum Institute, Denmark), which was defined as having 1,000 U/ml of C4 deposition activity, was used to generate a standard curve on mannan-coated wells in order to obtain relative C4 deposition activity.

Thrombin-like activity was assayed using 384 well plates, which were coated with IAV as above. After washing, the wells were incubated with 10% serum diluted in the binding buffer. After washing again, wells were incubated with rhodamine 110-thrombin substrate (R22124, Invitrogen, CA) in TBS-CaCl_2 _and read using 500 nm excitation/520 nm emission using the SpectraMax M5.

### Mice

MBL null mice were generated and fully backcrossed onto C57Black/6J [23,25]. Mice were used at ages between 6 and 10 weeks old. Gender and age were matched in each experiment. All animal experiments were performed under a protocol approved by the Subcommittee on Research Animal Care at Massachusetts General Hospital, Boston, MA.

### MBL detection in bronchoalveolar lavage fluid (BALF)

Mice were euthanized and a 22G catheter was inserted into the bronchi and secured with a nylon suture (6-0, Ethicon). BALF was collected using 3 lavages with 0.5 ml of PBS-EDTA and combined (Recovered BALF was approximately 1 ml). After centrifugation, four fifths of the BALF was mixed with TBS, supplemented with 10 mM CaCl_2 _and 1 M NaCl (binding buffer) and incubated with 20 μl of mannose agarose beads (Sigma, 1:1 in the binding buffer) over night on an end-over-end rotator at 4°C. The mannose agarose beads were collected and washed with TBS. The washed beads were subjected to SDS-PAGE using a 12% polyacrylamide gel under reducing conditions. Fractionated proteins were transferred to a nylon membrane (Immobilon P, Millipore) and Western blot analysis was performed using rabbit anti-human MBL Ab [26]. 1 μg of purified native mouse serum MBL was used as a positive control. The reaction was visualized using Western blue (Promega, WI). MBL bands were analyzed using a ChemiDoc scanner (Bio-Rad, CA) and the software provided with the ChemiDoc. MBL amounts were calculated as relative % volume of 100%, which combined all MBL bands in WT, MBL-A null, MBL-C null and the purified native mouse serum MBL.

MBL ELISA of the BALF was performed using previously described methods with minor modifications [27]. Briefly, 96 well plates were coated with mannan. Following washing and blocking, the wells were incubated with diluted BALF, 50% in the binding buffer. Bound MBLs were detected by rat monoclonal antibodies against MBL-A or MBL-C followed by alkaline-phosphatase conjugated anti-rat Ab and pNTP substrate. The reactions were read at 415 nm using the SpectraMax M5.

### Viral infection-induced cell death assay

Peritoneal resident MΦs were prepared by lavage of the peritoneal cavity with 5 ml PBS, performed twice for each animal and pooled. Lavaged peritoneal cells were washed and plated at 4 × 10^4 ^cells/well in 50 μl of culture media (RPMI1640, supplemented with 10% FBS). After incubation for 1 hr at 37°C, 5% CO_2_, wells were washed with PBS to remove non-adherent (non-MΦ) cells. Adherent cells (MΦs) were further incubated with influenza A virus (5 × 10^6 ^ffc/40 μl/well in RPMI1640) at 37°C in a CO_2 _incubator for 1 hr. 60 μl of culture media was added and incubated over night at 37°C in a CO_2 _incubator. Cell survival was assayed using WST2 reagent (Dojindo Molecular technologies, Inc., MD), according to the manufacturer's instruction. The WST2 reaction was read at OD 450 nm using the Spectramax M5. Cell death (%) was calculated by the formula: [OD 450 nm of WT MΦs alone - OD 450 nm of test groups) × 100)/OD 450 nm of WT MΦs alone].

### In vivo IAV infection experiment

IAV, Philippine 82 H_3_N_2 _(Phil82) and Phil82BS, which lacks one glycosylation site compared to Phil 82, were prepared as described previously [28]. Mice were anesthetized with avertin as described previously and were intranasally inoculated with 5 × 10^6 ^fluorescent foci counts (FFC) of IAV in 50 μl PBS. Bronchoalveolar lavage fluid (BALF), BAL cells and lung homogenates were prepared on days 1, 4 and 7 following virus infection as described previously with minor modifications [29]. BALF aliquots were stored in the -80°C freezer for use in the experiments on soluble factors. BAL cells were spun on to individual glass slides using a Cytopro centrifuge (Wescor Inc., UT) and stained using Diff-Quick (Sigma, MO) for differential cell counts under a microscope (Nikon 800). Apoptotic cells were identified by positive staining for Annexin V and counterstaining with Hoechst stain for cell type identification. A total of 100 ~ 120 cells in 3 ~ 5 fields per sample were counted in a blinded manner, in which samples were labeled with only mouse identification numbers.

Virus titers were determined using a MDCK infection assay as previously described [24]. Reconstitution experiments using MBL null mice and rhMBL were performed by intraperitoneal injection of 75 μg rhMBL (a gift from Enzon, USA) at one hr prior to viral infection, as previously described [23]. The 75 μg dose was calculated based on the lectin complement activation activity of rhMBL and purified mouse MBLs [23]. In addition, restored lectin pathway activity in MBL null mice was similar to that in WT mice [23].

### Assay of soluble molecules in BALF of IAV-infected mice

BALF on day 1 post-viral infection was collected as described above. Three BALF aliquots were pooled and were analyzed for 62 soluble molecules in duplicate using a cytokine antibody array (RayBiotech Inc., GA), according to the manufacturer's instructions and as previously described [25]. Chemiluminescence reaction in membranes was simultaneously captured by the ChemiDoc (Bio-Rad, Hercules, CA) and relative chemiluminescent intensity (arbitrary units) was obtained using the software provided with the ChemiDoc. Results were expressed as the fold-increase in WT relative to MBL null mice or vise versa. 2-fold increase was defined as positive.

### Statistical analysis

All data were analyzed by ANOVA or Wilcoxon/Kruskal-Wallis tests for non-parametric data using JMP software (SAS institute Inc., NC). p values less than 0.05 were considered to be significant.

## Results

### Human and murine MBL binds and neutralizes influenza A viruses

**Figure 1:**
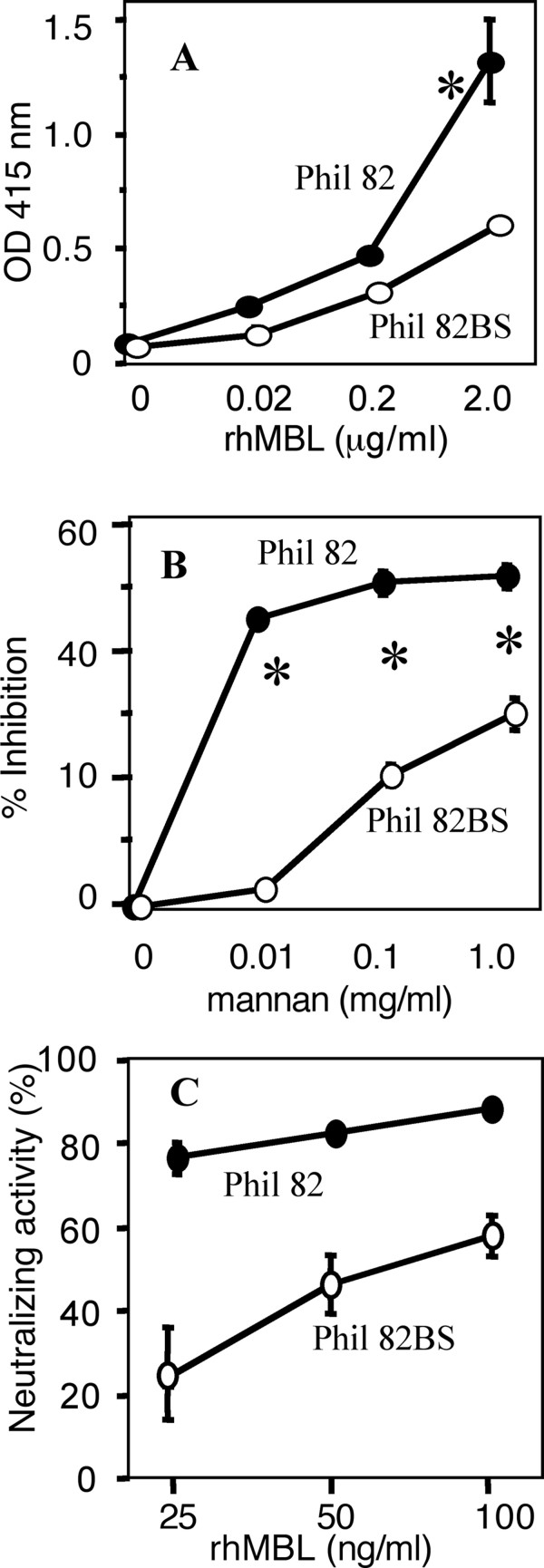
**Recombinant human MBL (rhMBL) binds and neutralizes IAV**. Closed circles and open circles represent Phil82 and Phil82BS strain, respectively. A, rhMBL binding to IAV. B, Mannan inhibition of rhMBL-IAV binding. Both assays were performed in triplicates and expressed as mean ± SD. C, Neutralizing activity of rhMBL. Assay was performed in duplicates and repeated twice. All data was combined and expressed as mean ± SE. *, p < 0.05.

We chose Phil82 and Phil82 BS strains because the latter lacks one glycosylation site from the parent Phil82 strain [28], thus it was hypothesized that MBL should bind Phil82 more efficiently compared with Phil82BS. As expected, MBL bound to Phil82 significantly greater than Phil82BS (Figure 1A). Exogenous mannan abolished MBL-binding more efficiently against Phil82 compared to Phil82BS (Figure 1B), suggesting that the virus binding was mediated through the CRD of MBL. Virus neutralizing activity was correlated with IAV-MBL binding activity (Figure 1C). This result supports the previous finding that rhMBL alone is able to neutralize IAV [10]. These results demonstrate that MBL by itself is capable of inhibiting IAV infection and that the activity is MBL-binding dependent.

**Figure 2:**
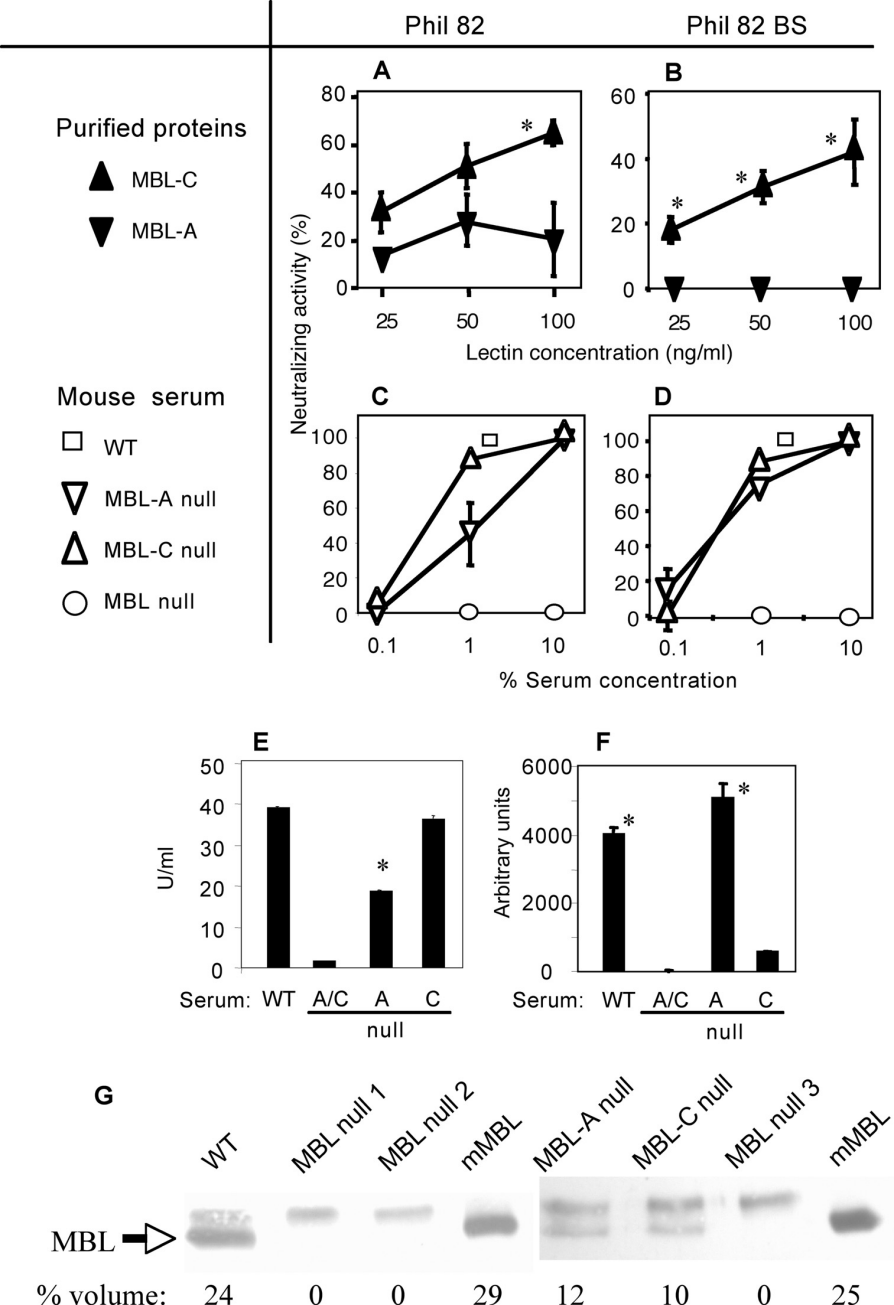
**Murine MBL-A and MBL-C**. A and B, Viral neutralizing activity of purified native MBL-A and MBL-C against Phil82 (A) and Phil82BS (B). C and D, Viral neutralizing activity of mouse serum against Phil82 (C) and Phil82BS (D). Experiments were repeated at least twice. All data were combined and expressed as mean ± SE. *, p < 0.05. E, Lectin complement pathway activation activity. C4 deposition on virus was expressed as U/ml. Assays were performed in duplicate. WT, wild type; A/C, MBL-A/MBL-C null (= MBL null), A, MBL-A null, C, MBL-C null. Representative data of three experiments is shown. Assays were performed in triplicate and expressed as mean ± SE. *, p < 0.0001 against WT and MBL-C null. F, Thrombin-like activity. Same serum source as in Figure 2E. Assays were performed in triplicate and expressed as mean ± SE. *, p < 0.0001 against MBL null and MBL-C null. G, Presence of MBL in lungs. Western blot analysis of affinity purified MBL from BALF. mMBL, purified native serum murine MBL (1 μg) as a positive control. Each lane represents individual mouse. % volume for detected MBL bands was calculated as described in the materials and methods.

Next, we assessed the viral neutralizing activity of murine MBL-A and MBL-C. Purified MBL-C demonstrated greater viral neutralizing activity against Phil82 than purified native MBL-A (Figures 2A and 2B). Further experiment showed no inhibitory effect of the purified MBL-A even at 400 ng/ml. The concentration used in this study, 100 ng/ml, is comparable to the MBL concentration that was detected in BALF following viral infection in mice [18]. Purified MBL-C was also able to inhibit Phil82BS IAV (Figure 2B).

We subsequently tested the effect of sera from various mouse strains in a similar manner. MBL-A deficient serum (= MBL-C sufficient) and MBL-C deficient serum (= MBL-A sufficient) demonstrated similar viral neutralizing activity to both viral strains (Figures 2C and 2D). The activity was observed at 1% and 10% serum but not at 0.1% serum. Thus, despite the undetectable direct viral neutralizing activity of MBL-A against Phil82BS (Figure 2B) the serum containing MBL-A (= MBL-C null serum) demonstrated IAV neutralizing activity. The serum concentration of MBL-A and MBL-C is approximately 10 and 25 μg/ml, respectively, as we previously assayed in these mice (57). Therefore, the concentration of MBL-A and MBL-C in 1% serum is 100 ng/ml and 250 ng/ml, respectively, which are comparable to those tested for purified MBL proteins.

Strikingly, serum lacking both MBL (MBL null) lost more than 50% of the viral neutralizing activity compared with WT serum and serum lacking MBL-A or MBL-C against both viral strains (Figures 2C and 2D). Taken together, these data suggested that IAV neutralizing activity was MBL-dependent and that serum factors augmented MBL's viral neutralizing activity.

### MBL activates complement and a thrombin-like activity on IAV

Lectins activate complement and coagulation as an antimicrobial mechanism [12-14]. Therefore, we investigated MBL-mediated activation of complement and a thrombin-like activity against IAV. The lectin complement pathway activity was comparable between MBL-C null (MBL-A sufficient) and WT mouse serum while the activity was about one-half in MBL-A null serum (MBL-C sufficient) and was negligible in MBL null serum (Figure 2E). These results support our previous findings that purified native MBL-A activated the lectin complement pathway more effectively than purified native MBL-C on a mannan-coated surface [23].

In contrast, thrombin-like activity was observed in WT and MBL-A null mouse serum at comparable levels while it was only one-tenth in MBL-C null mouse serum and was undetectable in MBL null mouse serum (Figure 2F). These data suggest that MBL-A and MBL-C preferentially activate the lectin complement pathway and thrombin-like activity, respectively. These MBL-mediated activities were results of activated MASPs, which can bind MBL [11].

### Presence of MBL in the lung

In order to determine presence of MBL in the lung, D-mannose agarose beads, to which MBL has high affinity, were incubated with BALF collected from each mouse and were then subjected to Western blot analysis. Two bands were observed. One of them was immediately above mMBL bands and was absent in the purified mMBL (mMBL, 35 kD). Therefore, these bands were concluded to be due to non-specific reaction of the rabbit anti-human MBL polyclonal Ab. Protein bands, corresponding to purified mMBL, were detected in BALF of WT mice whereas they were undetectable in MBL null mice (Figure 2G). Relative % volume of the MBL band in WT mouse BALF was 24 and was close to 29 of mMBL 1 μg (Figure 2G). As expected, the relative %volume of MBL bands in MBL-A or MBL-C single null mouse BALF was 12 and 10, respectively, therefore these were roughly 50% of MBL in WT mouse BALF, which contains both MBL-A and MBL-C (Figure 2G). These Western blot analyses demonstrated that approximately 1 μg of combined MBL-A and MBL-C was present in the resting healthy lung of WT mice. However, ELISA using aliquots of the same BALF sample was unable to detect MBL. This may be due to the ELISA being insufficiently sensitive, or possibly related to other factors in the BALF. The finding of MBL in the resting lung supports our idea that MBL has a role in the lung in preventing IAV.

### Increased viral infection in MBL null mice

**Figure 3:**
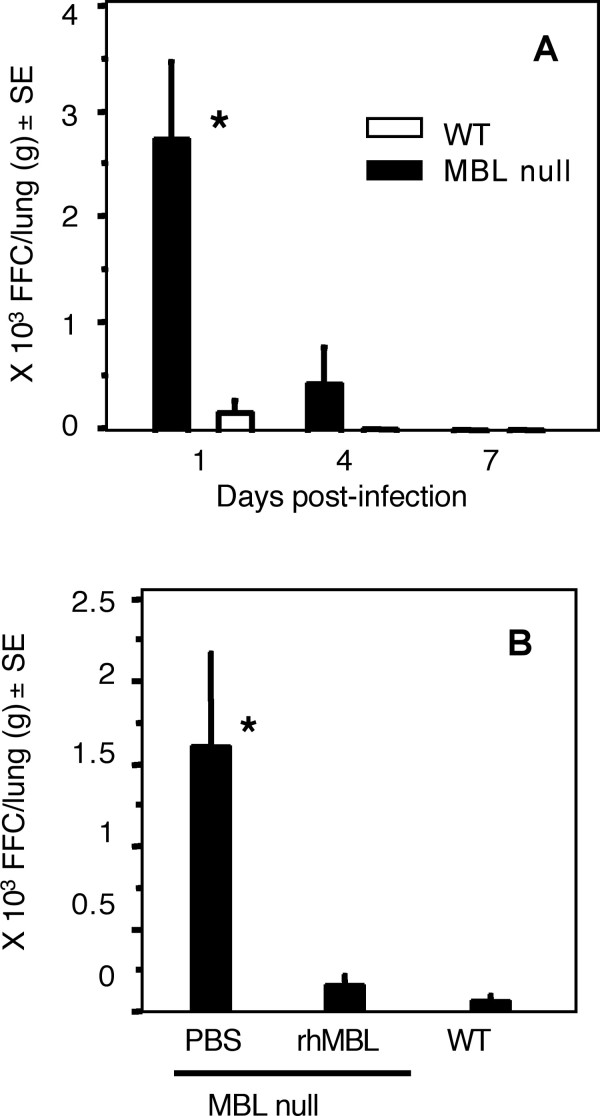
**Increased virus titers in the lungs of MBL null mice**. A, Virus titers in lung homogenates following IAV infection were assayed on days 1, 4 and 7. Three mice were used for each group at each time point. Virus titers were expressed as FFC/lung (g) ± SE. *, p < 0.005. B, Administration of rhMBL rescues susceptible phenotype of MBL null mice. MBL null mice were reconstituted with rhMBL or PBS as a control and virus titer was determined in lung homogenates at 24 hr after virus inoculation. Mice used were 7, 5 and 4 for MBL null, MBL null + rhMBL and WT mice, respectively. Virus titers were expressed as FFC/lung (g) ± SE. *, p < 0.005.

To test our hypothesis that MBL prevents IAV infection, we subjected MBL null and WT mice to primary lung infection with IAV. For *in vivo *study, we chose Phil82 strain because both Phil82 and Phil82BS strains were similarly neutralized by MBL containing mouse sera despite the difference in viral-MBL binding capacity (Figures 2A, B, C, and 2D). Thus, we concluded that *in vivo *responses against Phil82 strain would be similar to those against Phil82BS. Virus was not detected in lungs following intranasal inoculation of PBS in both WT and MBL null mice (data not shown). In contrast, viral titers in lungs of MBL null mice were significantly higher compared with WT mice on day 1, after which they decreased to low to undetectable at later time points, days 4 and 7 in both MBL null and WT mice (Figure 3A). These results demonstrate that MBL null mice have an increased susceptibility to IAV infection, suggesting that lack of MBL reduces the host defense against IAV in the lung.

To further explore these findings, MBL null mice were injected (i.p.) with 75 μg of rhMBL one hr prior to viral inoculation [23]. Virus titers in lungs of reconstituted MBL null mice were comparable to that of WT mice (Figure 3B). These results confirmed that the increased susceptibility to IAV infection in MBL null mice was due to the lack of MBL and that MBL deficiency could be corrected by administration of rhMBL.

### Increased total WBCs in BAL of WT mice

**Figure 4:**
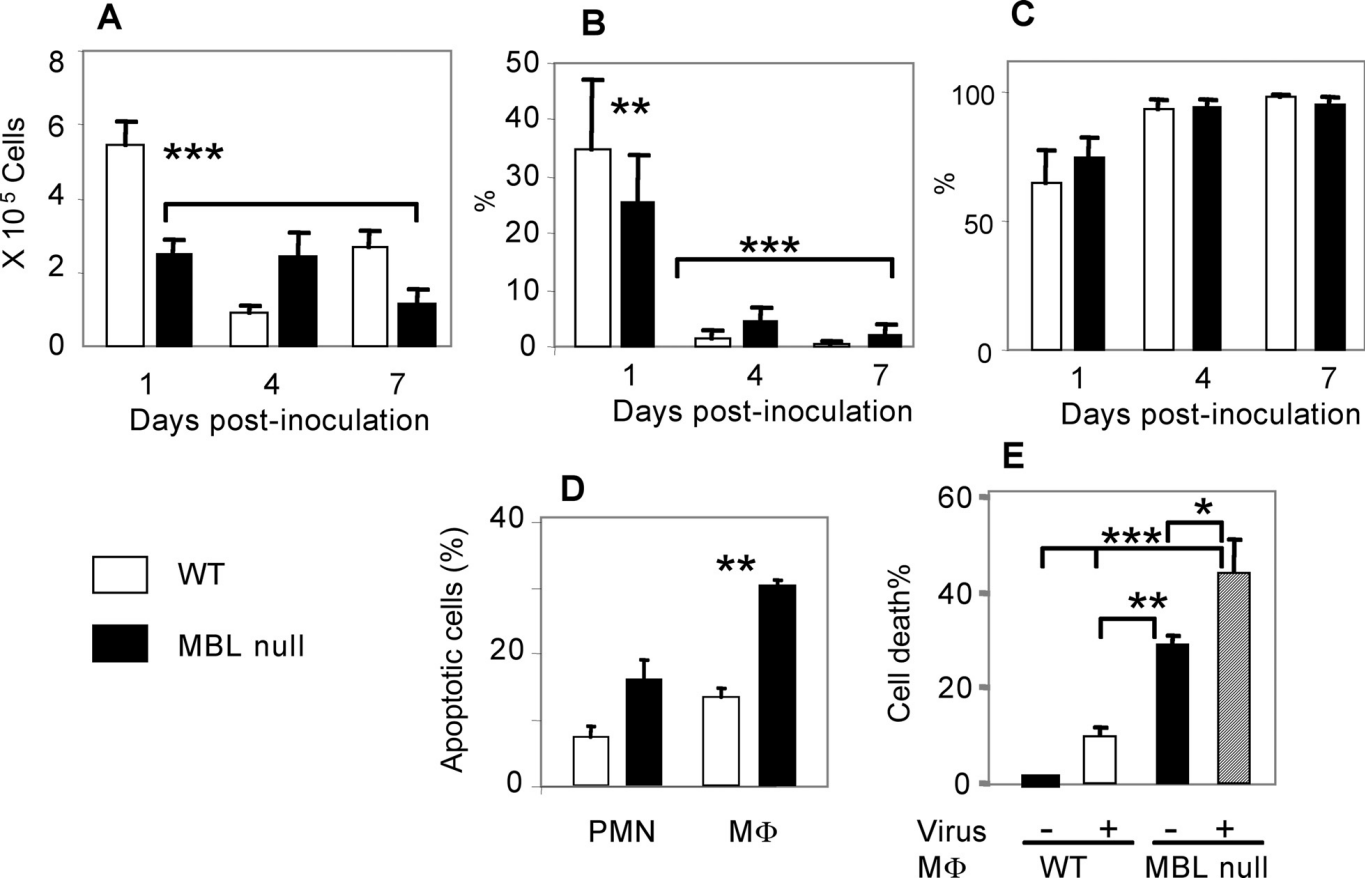
**White blood cells (WBC) and apoptosis susceptibility**. A, Total WBC are expressed as cells per lung. B, PMN population as % of total WBC. C, Macrophage population as % of total WBC. For all experiments A - C, two experiments were combined. Mice numbers used were 5 wild type (WT) and 7 MBL null for day 1; 6 WT and 7 MBL null for day 4; and 8 WT and 7 MBL null for day 7. Data are expressed as mean ± SE. **, p < 0.005 and ***, p < 0.001. D, Increased apoptotic macrophages (%) in BAL cells of MBL null mice on day 1 post-IAV infection. Experiments were as in Figure 4A. Five wild type (WT) and 7 MBL null mice were used. Data are expressed as mean ± SE. **, p < 0.005. E, Viral infection induced cell death. Macrophages (MΦ) from WT and MBL null mice were incubated with virus and cell death was expressed as % of WT MΦ without viral infection. Assays were performed in triplicate. Data are expressed as mean ± SE. *, p < 0.05; **, p < 0.005; and ***, p < 0.0001.

Next, we examined BAL cells in the infected lungs. Total WBCs in BAL of WT mice were significantly increased compared with MBL null mice on day 1 while they were similar in both strains of mice at the later time points (Figure 4A). Of these WBCs, the PMN population was significantly increased in both WT and MBL null mice at day 1 compared with the later time points. Furthermore, significantly more PMNs were observed in the lungs of WT mice compared with MBL null mice on day 1 (Figure 4B). This PMN influx was caused by the viral infection because no PMN was observed in the lungs of naïve MBL null and WT mice (data not shown). We observed only MΦs in the lung of naïve MBL null and WT mice, and MΦs were also the predominant cell type at all time points during viral infection (Figure 4C). These data suggested that MBL or the effect of MBL/MASP activation was involved with recruitment of WBCs, and in particular PMNs, into the infected alveolar space during viral infection.

### *MΦs *of MBL null mice are prone to apoptosis

Viral infection is known to generate a large amount of apoptotic cells [30]. Therefore, we looked for apoptotic cells in the lungs of MBL null and WT mice on day 1 following IAV infection. Apoptotic MΦs were significantly increased in MBL null mice compared with WT mice (Figure 4D). Although apoptotic PMNs were also increased in MBL null mice compared with WT mice this difference was not statistically significant (Figure 4D).

We subsequently assessed IAV infection-associated cell death of MΦs isolated from WT and MBL null mice. Resident peritoneal MΦs were prepared simultaneously and were infected with IAV. Unexpectedly, MΦs from MBL null mice had significantly higher cell death compared with even IAV-infected WT MΦs (Figure 4E). Further, IAV infection increased cell death of MBL null MΦs (Figure 4E). In contrast, WT MΦ did not show significantly increased cell death even after IAV infection (Figure 4E).

### Soluble factors in BALF following viral infection

**Figure 5:**
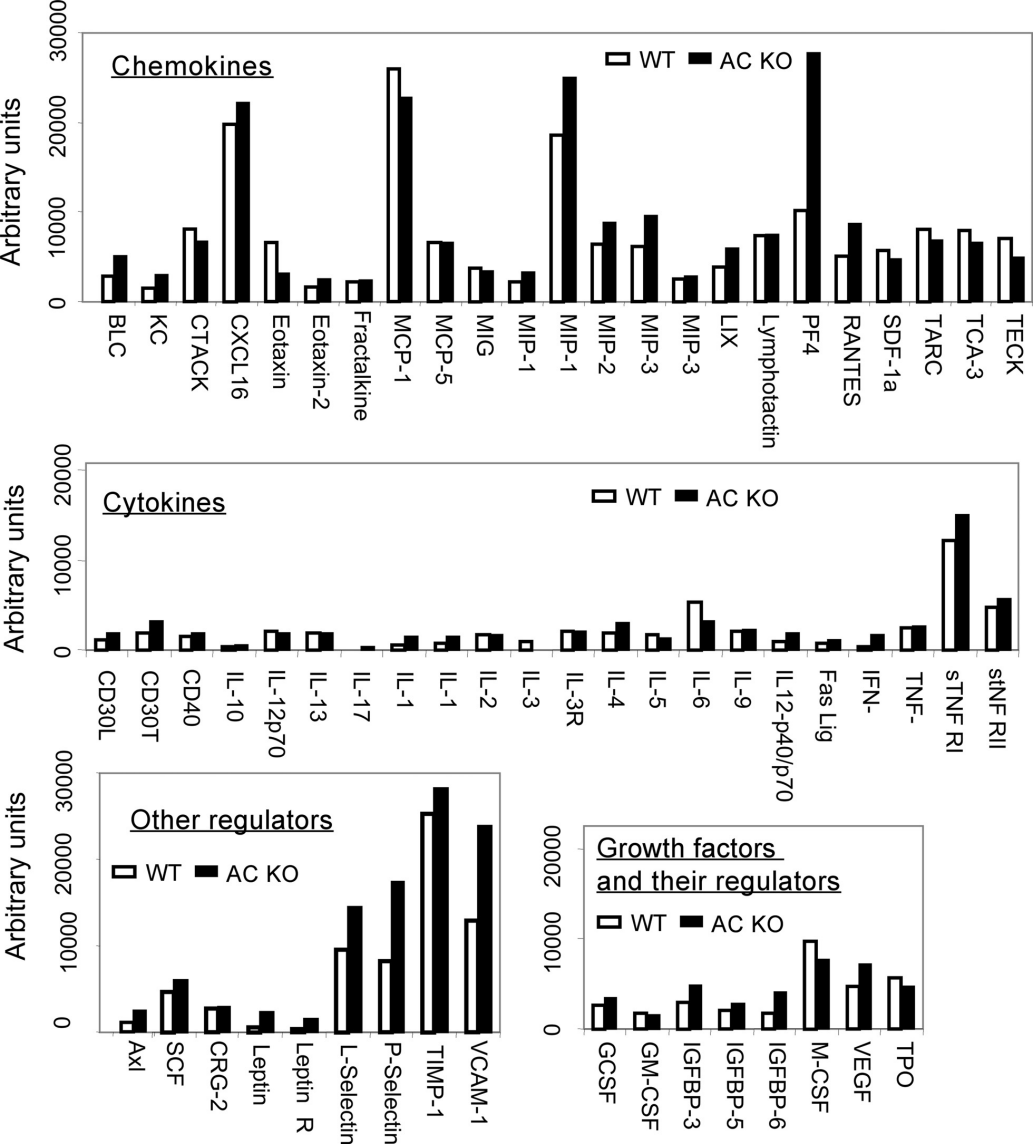
**Biological responses in bronchoalveolar fluid during IAV infection**. Protein array experiments were performed on BALF from day 1 post-viral infection. BALF from 3 mice in each group were pooled. Arbitrary units of each molecule for WT and MBL null mice are shown. Data are average of duplicates.

**Figure 6:**
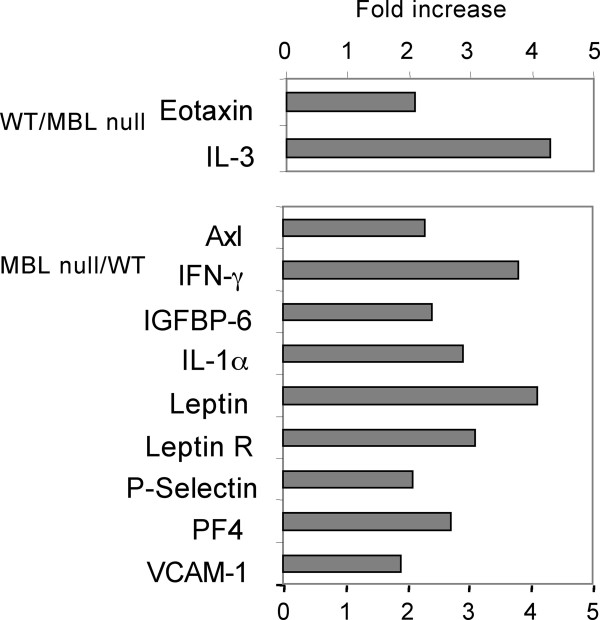
**Molecules increased more than two fold, either wild type to MBL null (WT/MBL null) or reverse (MBL null/WT), based on the results in Figure 5**.

We analyzed BALF for 62 soluble molecules during viral infection using multiple factor assay kits. We focused on day 1 post-viral infection because this time point demonstrated a significant difference for viral titer and WBC responses between WT and MBL null mice as we described above. Ten molecules, CXCL16, MCP-1, MIP-1γ, PF4, sTNF RI, L-selectin, P-selectin, TIMP-1, VCAM-1, and M-CSF increased to more than 10,000 units (Figure 5) (additional file 1). However, expression level of these molecules was similar between WT and MBL null mice except for platelet factor 4 (PF4) and vascular cellular adhesion molecule-1 (VCAM-1), which increased more than 2-fold in the BALF of MBL null mice compared with WT mice. Similarly, 7 other molecules were also increased in the BALF of MBL null mice. These molecules included cytokines (IFN-γ and IL-1α); adhesion molecules (P-selectin); and other regulatory molecules (Axl tyrosine kinase, insulin-like growth factor binding protein-6 (IGFBP-6), Leptin, and Leptin receptor) (Figure 6). In contrast, only two molecules, eotaxine (CCL 11) and IL-3 were increased more than 2-fold in WT mice compared with MBL null mice (Figure 6). These results suggested that MBL modulated inflammation during IAV infection in the lung leading to a numbers of changes in the balance of regulatory molecules.

## Discussion and Conclusion

Our results provide the first *in vivo *evidence that MBL deficiency increases susceptibility to IAV infection. Importantly, the increased infection susceptibility can be improved with rhMBL, as administration of rhMBL to MBL null mice reduced viral infection similar to WT levels. This study also has revealed the presence of MBL in the healthy resting lung. We used affinity purification to isolate MBL from BALF. This procedure was clearly more sensitive than ELISA, in which MBL was detected only after infection [18]. In the previous study, MBL in the BALF was measured by ELISA, which further dilutes protein concentrations in addition to the initial dilution from the fluid used to perform lung lavage [18]. We confirmed that ELISA did not detect MBL in un-concentrated BALF that were also used in the previous study [18]. These results suggest that MBL, a serum protein, most likely leaks into the alveolar space and that MBL also participates in innate immune protection against infection in the lung.

This study demonstrates that MBL inhibits viral infection directly as well as indirectly with cooperation of serum factors. These findings support previous studies showing that MBL directly neutralizes and inhibits IAV infection and that there are direct [10] and indirect anti-viral activities, including involvement of complement [31]. Of interest, we show that Phil82BS, which lacks one glycosylation site from the parent strain Phil82, also activates the lectin complement pathway and thrombin-like activity despite reduced MBL binding. Robust complement activation despite reduced binding of MBL to Phil82BS could be explained by recent findings that the lectin pathway activity is amplified by the alternative pathway, suggesting that even a lower degree of binding may be sufficient in inducing effective anti-viral activity [32,33].

The sugar specificity of MBL-A and MBL-C is slightly different and MBL-A is an acute phase protein while the expression of MBL-C is not influenced to the same extent by inflammatory stimuli [27]. We now show that MBL-C is more effective in direct anti-viral activity than MBL-A *in vitro*. This is the first observation demonstrating a difference between MBL-A and MBL-C in inhibiting a pathogen. This difference is diminished by co-operation of serum factors, since MBL-C deficient serum, which is MBL-A sufficient, is as effective as MBL-A deficient serum (MBL-C sufficient) at neutralizing IAV. This cannot be attributed to an increase of MBL-A in MBL-C null mice because our previous study demonstrates that MBL-A in MBL-C null mice is similar to that in WT mice and vice versa [23]. Importantly, these serum-facilitated anti-viral activities are initiated by MBL because MBL null mice serum does not show viral neutralizing activity even at high concentration.

MBL-ligand binding induces conformational changes in MASPs, resulting in activated serine proteases. Surprisingly, thrombin-like activity is mediated by MBL-C whereas the lectin complement pathway is more efficiently mediated by MBL-A (supporting our previous findings [23]). These data suggest that direct anti-viral activity of MBL-C correlates with thrombin-like activity. Interestingly, human MBL is genetically homologous to MBL-C and also mediates thrombin-like activity [13]. These findings raise the possibility that, in addition to mediating complement activation, MBL may contribute to host defense by activating coagulation. Hence, complement and coagulation activity may be effective innate immune mechanisms not only in primitive animals, like the horseshoe crab, [14] but also in mammals.

Our study also demonstrates that MBL modulates cellular responses, increasing recruitment of WBCs, and in particular PMNs, which we have shown mediate viral clearance [34], although the overall predominant cell type is MΦ in both WT and MBL null mice. A marked increase of apoptotic cells was observed in MBL null mice during IAV infection. This result could be explained, in part, by reduced clearance of apoptotic cells, as MBL null mice have impaired apoptotic cell removal [15]. An unexpected finding is that MΦs of MBL null mice seem to be susceptible to apoptosis once these are isolated and placed *in vitro*.

Pathogenesis of IAV infection has been linked to polymerase basic (PB)1-F2, which induces apoptosis upon infection [35], suggesting that viral infection induces host cell apoptosis to minimize host cellular responses to the virus. In this scenario, prevention of apoptosis is a host defense mechanism. Taken together, these observations suggest that immune cells of MBL deficient hosts are more easily infected and more prone to apoptosis, and that impaired clearance of apoptotic cells would further increase the burden of infection.

Multifactorial high throughput assays of BALF have revealed that the lung of WT mice is relatively quiescent compared with that of MBL null mice, because only two molecules, IL-3 and eotaxin were increased following IAV infection. Although IL-3, a mast cell growth factor, has been linked to lung diseases in animal studies [36], mast cells themselves have been known to play a role in wound healing [37]. Eotaxin (CCL11) has been identified in lung tissue repair as a chemo-attractant of airway smooth muscle and as a lung fibroblast growth factor [38]. These observations suggest that the lungs of WT mice are in the wound-healing phase as early as on day 1 after IAV infection. In contrast, the lungs of MBL null mice have increases of 9 molecules: Leptin, Leptin receptor, IFN-γ, IL-1α, PF4, IGFBP-6, P-selectin, VCAM, and Axl tyrosine kinase. Surprisingly, all these molecules have been associated with and/or attributed to lung injuries [39-48], suggesting that MBL deficient hosts may be prone to tissue damage from infection. Moreover, these factors may also contribute to increased susceptibility to apoptosis of MBL null macrophages, as discussed above. Taken together, these observations suggest that MBL plays a role in preventing tissue injury, and further study is required to elucidate the details of these processes.

It is important to note that even MBL deficient mice cleared virus by day 4 in our study. The likelihood is that lung-surfactant proteins are contributing to anti-viral activity, as SP-A and SP-D are synthesized in the lung and possess anti-viral activities, including neutralization, opsonization and hemagglutination-inhibition of virus [17]. Mice lacking SP-A or SP-D are susceptible to IAV infection [17]. Although SP-A, SP-D and MBL belong to the collectin family, the surfactant proteins do not activate complement, contrast to MBL [15]. Surfactant proteins do not seem to form a complex with complement activating serine proteases, such as MASPs, and most likely do not activate coagulation. In contrast to these differences, these three collectins do influence adaptive immunity [49-51] although their influence and the details of their actions against IAV are not well understood. Taken together, these observations suggest that collectins may function cooperatively together to eliminate virus. Further studies are warranted to elucidate the details of the interaction among these collectins.

In conclusion, our study demonstrates *in vivo *evidence that MBL protects hosts from IAV infection and that MBL may be a new useful adjunctive anti-IAV therapy. Anti-IAV mechanisms include activation of the lectin complement pathway and of coagulation through a thrombin-like activity, both of which are innate immune mechanisms. Our investigation also suggests that MBL deficiency may be a risk factor for IAV infection. Thus, MBL, as an element of the innate immune system, plays an important role in protecting and maintaining lung homeostasis.

## Abbreviations used

BALF: bronchoalveolar lavage fluid; CRD: carbohydrate recognition domain; IAV: influenza A virus; IGFBP-6: insulin-like growth factor binding protein-6; FFC: fluorescent foci counts; MΦs: macrophages; MCP-1: macrophage chemotactic protein-1; MDCK: Madin-Darby canine kidney; MIP-1γ: macrophage migration inhibitory protein-1γ; MBL: mannose-binding lectin; MASP: MBL-associated serine protease protease; PF4: platelet factor 4; PMN: polymorphonuclear neutrophil; rhMBL: recombinant human MBL; SP-A: surfactant protein-A; SP-D: surfactant protein D; TIMP-1: tissue inhibitor of metalloproteinase-1; VCAM-1: vascular cell adhesion molecule-1; WBC: white blood cell; WT: wild type.

## Authors' contributions

WC performed in vitro assays. MRW and KLH provided IAV and performed in vitro assays and viral titration. PM and SM assisted mice breeding and experimental procedures. ST purified mouse MBLs and provided anti-MBL antibody. KT performed *in vivo *mouse studies and performed *in vitro *assays and oversaw the entire project. All authors contribute preparation of the manuscript.

## Supplementary Material

Additional file 1 **Protein array data**. Raw data of the protein array and a protein map. Click here for file
