# Supplementary material for: Lack of the pattern recognition molecule mannose-binding lectin increases susceptibility to influenza A virus infection
Source: BMC Immunol. 2010 Dec 23;11:64. doi: 10.1186/1471-2172-11-64 (PMC3022599; doi:10.1186/1471-2172-11-64)
Supplement: Additional file 1 — Protein array data. Raw data of the protein array and a protein map. [file 1471-2172-11-64-S1.PDF]

Additional file 1. Developed protein array membranes and the protein map. Three mice in each group were pooled.

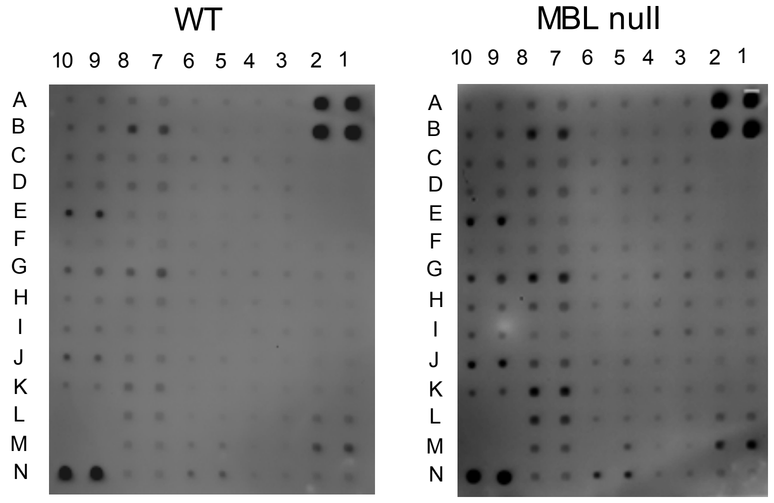


| | 10 9 | 8 7 | 6 5 | 4 3 | 2 1 |
|---|---|---|---|---|---|
| A | SDF-1a | Lymphotactin | IL-4 | Eotaxin-2 | Pos |
| B | TRAC | MCP-1 | IL-5 | Fas Ligand | Pos |
| C | TCA-3 | MCP-5 | IL-6 | Frctalkine | Neg |
| D | TECK | M-CSF | IL-9 | GCSF | Neg |
| E | TIMP-1 | MIG | IL-10 | GM-CSF | Blank |
| F | TNFa | MIP-1a | IL-1p40/p70 | IFN-g | Axl |
| G | sTNF R1 | MIP-1g | IL-12p70 | IGFBP-3 | BLC |
| H | sTNF RII | MIP-2 | IL-13 | IGFBP-5 | CD30L |
| I | TPO | MIP-3b | IL-17 | IGFBP-6 | CD30 T |
| J | VCAM-1 | MIP-3a | KC | IL-1a | CD40 |
| K | VEGF | PF-4 | Leptin R | IL-1b | CRG-2 |
| L | Blank | P-Selectin | Leptin | IL-2 | CTACK |
| M | Blank | RANTES | LIX | IL-3 | CXCL10 |
| N | Pos | SCF | L-Selectin | IL-3Eb | Eotaxin |
